# Functional and phylogenetic diversity determine woody productivity in a temperate forest

**DOI:** 10.1002/ece3.3857

**Published:** 2018-01-29

**Authors:** MinHui Hao, Chunyu Zhang, Xiuhai Zhao, Klaus von Gadow

**Affiliations:** ^1^ Research Center of Forest Management Engineering of State Forestry Administration Beijing Forestry University Beijing China; ^2^ Faculty of Forestry and Forest Ecology Georg‐August‐University Göttingen Göttingen Germany; ^3^ Department of Forest and Wood Science University of Stellenbosch Stellenbosch South Africa

**Keywords:** Biodiversity–productivity relationship, biomass, environmental conditions, functional diversity, phylogenetic diversity, structural equation models

## Abstract

Understanding the relationships between biodiversity and ecosystem productivity has become a central issue in ecology and conservation biology studies, particularly when these relationships are connected with global climate change and species extinction. However, which facets of biodiversity (i.e. taxonomic, functional, and phylogenetic diversity) account most for variations in productivity are still not understood very well. This is especially true with regard to temperate forest ecosystems. In this study, we used a dataset from a stem‐mapped permanent forest plot in northeastern China exploring the relationships between biodiversity and productivity at different spatial scales (20 × 20 m; 40 × 40 m; and 60 × 60 m). The influence of specific environmental conditions (topographic conditions) and stand maturity (expressed by initial stand volume and biomass) were taken into account using the multivariate approach known as structural equation models. The variable “Biodiversity” includes taxonomic (Shannon), functional (FDis), and phylogenetic diversity (PD). Biodiversity–productivity relationships varied with the spatial scales. At the scale of 20 × 20 m, PD and FDis significantly affected forest biomass productivity, while Shannon had only indirect effects. At the 40 × 40 m and 60 × 60 m scales, biodiversity and productivity were weakly correlated. The initial stand volume and biomass were the most important drivers of forest productivity. The local environmental conditions significantly influenced the stand volume, biomass, biodiversity, and productivity. The results highlight the scale dependency of the relationships between forest biodiversity and productivity. The positive role of biodiversity in facilitating forest productivity was confirmed at the smaller scales. Our findings emphasize the fundamental role of environmental conditions in determining forest ecosystem performances. The results of this study provide a better understanding of the underlying ecological processes that influence specific forest biodiversity and productivity relationships.

## 1 INTRODUCTION

The study of the relationships between biodiversity and certain ecosystem functions has emerged as a central issue in ecology and conservational biology, particularly in connection with specific global scenarios involving the continuing extinction of species, as well as the increasing threats posed by climate changes (Cardinale et al., 2012; Loreau et al., 2001; Zhang & Chen, 2015). Biodiversity loss may lead to changes in ecosystem functions such as productivity, resilience, and nutrient cycling, due to the fact that sets of species with particular functional attributes may have been lost or replaced by others with different attributes (Balvanera et al., 2006; Cardinale et al., 2012). A great number of studies have been conducted involving the relationships between biodiversity and ecosystem functions. Several of these studies have reported positive results, and two hypotheses have been proposed: the niche complementarity hypothesis (Tilman et al., 1997) and the sampling effect hypothesis (Grime, 1998). Based on the niche complementarity hypothesis, the ecosystem functions are determined by niche partitioning and interspecific facilitation (Chiang et al., 2016; Mulder, Uliassi, & Doak, 2001; Tilman et al., 1997). However, based on the sampling effect hypothesis, the ecosystem functions are determined by the most dominant species, which are characterized by extraordinary traits and high productivity (Finegan et al., 2015; Grime, 1998; Ratcliffe et al., 2016). In addition to these positive relationships, unimodal, negative, or even insignificant relationships were found in both forest and grassland ecosystems (Healy, Gotelli, & Potvin, 2008; Srivastava & Vellend, 2005; Vilà, Vayreda, Gracia, & Ibáñez 2003).

One of the most intensely debated questions in the field of biodiversity–ecosystem function relationships is whether purely taxon‐based diversity indices, which neglect the function dissimilarity and evolutionarily relatedness of species, such as species richness or Shannon Index, can appropriately assess the biodiversity of a community (Cadotte, Cardinale, & Oakley, 2008; Laliberté & Legendre, 2010; Mokany, Ash, & Roxburgh, 2008; Mouchet, Villéger, Mason, & Mouillot, 2010).

Due to the limitations of taxon‐based diversity in evaluating biodiversity–ecosystem function relationships, several useful tools relating to the use of functional traits have been put forward (Laliberté & Legendre, 2010; Mokany et al., 2008; Mouchet et al., 2010). Functional traits refer to certain ecological, physiological, or morphological characteristics which are known to be important for plant growth, survival, and mortality, as well as for ecosystem functioning (Finegan et al., 2015; Lohbeck et al., 2012). Functional diversity (FD) refers to the value, range, distribution, or dispersion of the functional traits in a plant community (Díaz et al., 2011; Laliberté & Legendre, 2010; Mouchet et al., 2010; Villéger, Mason, & Mouillot, 2008). FD has been found to be more closely related to the functioning of ecosystems than species‐based diversity (Flynn, Mirotchnick, Jain, Palmer, & Naeem, 2011). This is mainly due to the fact that FD may increase niche complementarity through the efficient use of resources by the different species within a limited environment.

Phylogenetic diversity (PD) reflects the evolutionary history of a community (Webb, 2000; Webb, Ackerly, McPeek, & Donoghue, 2002). Previous studies have suggested that the PD could be used as a proxy of the FD, due to the hypothesis that evolutionary similarities may generate similar traits (Cadotte et al., 2008; Liu, Swenson, Zhang, & Ma, 2013; Srivastava, Cadotte, MacDonald, Marushia, & Mirotchnick, 2012). Moreover, when compared with FD, which is based on a finite set of traits, the expectation is that the PD may have a greater explanatory power. This is due to the fact that the PD potentially integrates a greater amount of trait information and represents a more inclusive overall measure of plant performance (Cadotte, Cavender‐Bares, Tilman, & Oakley, 2009; Cadotte, Hamilton, & Murray, 2009; Purschke et al., 2013). Therefore, it may be expected that the results of trait‐ or phylogeny‐based studies could potentially provide a better understanding of the biodiversity and ecosystem function relationships when compared with species‐based approaches. Although this conclusion may be largely based on the type of ecosystems where component species are functionally (and/or phylogenetically) similar or far apart, and opposite results have also been found (Venail et al., 2015).

Productivity and biomass are often used interchangeably in the grassland communities. However, in forest communities, biomass and productivity are distinctly different and should therefore be treated separately (Chisholm et al., 2013; Keeling & Phillips, 2007). Forest volume can be more conveniently measured than biomass (Bettinger, Boston, Siry, & Grebner, 2010). In a number of previous studies, the net increment in total volume was applied to measure forest productivity (Gadow & Hui, 1999; Liang et al., 2016). However, more recently, this approach has caused some controversy. For example, some researchers have argued that there is a potential risk in using volume to assess ecosystem functioning. This is due to the fact that volume does not take into account the differences in wood density, which may vary considerably among the species (Russell, Woodall, D'Amato, Domke, & Saatchi, 2014).

In the focusing on biodiversity–productivity relationships, bivariate analysis is one of the most frequently used approaches. However, an important issue with bivariate analysis is the fact that the relationships are usually rather complex and that the variations in diversity and productivity both may emerge from uncertain factors which will reduce the interpretative potential of the results. As a consequence, there have been increasing demands for more sophisticated statistical methods to evaluate these relationships. Structural equation model (SEM), as an integrative method, has been invoked to test such intricate relationships (Grace et al., 2016; Liu et al., 2016; Paquette & Messier, 2011; Zhang & Chen, 2015; Zhang, Chen, & Taylor, 2017). SEM is a powerful statistical approach for testing hypotheses about networks including direct and indirect causal relationships with a series of dependent and independent variables that may be correlated (Lamb, Mengersen, Stewart, Attanayake, & Siciliano, 2014). The SEM model has several advantages, including mathematical rigor, inferential capacity, flexibility for describing complex relationships between variables, and visually intuitive representation of networks among ecological factors (Lamb et al., 2014). According to a study which was carried out in 1,126 grassland plots spanning five continents, the SEM showed a higher explanatory power than bivariate analyses (Grace et al., 2016). It is not surprising that the bivariate analyses produced different results, as SEM differs from bivariate analyses in theorized cause–effect relationships among multiple processes and when the true causal pathways are more complex, bivariate analyses may be misleading. Based on these findings, SEM has the potential to do better at disentangling the complex relationships of biodiversity and productivity.

Biodiversity and productivity, along with their relationships, are jointly affected by a host of factors and processes. Using SEM, we can attempt to make reasonable and meaningful generalizations by simplifying the real ecosystems based on a multifactor research framework, which contains both biotic and abiotic factors such as stand maturity, soil and topographic conditions, and climate factors (Ali et al., 2016; Baker et al., 2009; Paquette & Messier, 2011; Ratcliffe et al., 2016; Russell et al., 2014; Zhang & Chen, 2015). Some of the previous studies took into account several of these factors. However, there is still plenty of work to do to clarify the intricate and interrelated relationships. For instance, many of the studies regarding the biodiversity–productivity relationships have often been criticized for failing to control the environmental variation. Environmental conditions can strongly influence the availabilities of water, light, and soil nutrients which are essential for plant growth. Environmental conditions are known to regulate plant traits and biodiversity patterns, as well as ecosystem productivity (Liu, Yunhong, & Slik, 2014; Zhang, Zhao, Zhao, & Gadow, 2012). In brief, environmental conditions have been proposed to be fundamental drivers, as the biodiversity–productivity relationships are shaped by environmental conditions through complex plant–soil feedback loops (Zhang et al., 2017). Moreover, some of the previous related studies have neglected the effects of spatial scale. In reality, the relationships between biodiversity and productivity should be scale‐dependent at the community level (Chisholm et al., 2013; Wang et al., 2016). This is due to the fact that forest productivity, measured as the annual biomass or volume increment per hectare, is expected to not change with spatial scale, whereas the species diversity would increase with the area of sampling. Finally, the characteristics of the stand itself (for example, the stand maturity) also play pivotal roles in determining the performance of a forest and should be included in the analysis framework (Zhang & Chen, 2015).

This study explores the complex relationships between biodiversity and productivity at different spatial scales simultaneously account for the influence of local environmental conditions, as well as the stand maturity. The observations of a 21.12 ha stem‐mapped permanent forest plot in northeastern China were used, including the information about tree growth and specific functional traits. Three measures of biodiversity were employed in this study. Productivity was expressed by the annual increments of stand volume or aboveground biomass. These variables were then used in SEMs in order to answer the following questions: (i) Which facets of biodiversity (species, phylogenetic, and functional diversity) have the greatest effect on forest productivity? (ii) How do the relationships between biodiversity and productivity vary with the spatial scale? and (iii) How are these relationships affected by the abiotic and biotic factors, specifically, the topographic variables and the initial stand biomass (or volume)?

## 2 MATERIALS AND METHODS

### 2.1 Study area and dataset

The study site is situated in the Jiaohe Management Bureau of the Forest Experimental Zone in the Jilin Province of northeastern China (43°57.897′–43°58.263′N, 127°42.789′–127°43.310′E; Figure S1). The mean annual temperature in this area is 3.8°; the average monthly temperature ranges from −18.6° in January to 21.7° in July. The mean annual precipitation is 695.9 mm. A permanent forest observational study covering an area of 21.12 ha (660 × 320 m) was established during the summer of 2009. The last recorded tree harvesting activities of this study plot took place more than 50 years ago, and now, it represents a middle‐to‐late stage of succession (Wang et al., 2016). The vegetation type is a mixed broadleaf–conifer temperate forest. The dominant species are *Juglans mandshurica*,* Acer mono*,* Tilia amurense*,* T. mandshurica*,* Fraxinus mandshurica*, and *Pinus koraiensis*. All of the woody stems within the study plot with diameters at breast height (DBH) which exceeded 1 cm were tagged, measured, and stem‐mapped, and their species were identified (Wang et al., 2016; Zhang et al., 2012). This plot was recensused during the summer of 2014.

In order to examine the relationships between the biodiversity and forest productivity along an environmental gradient at different spatial scales, the study area was subdivided into quadrats of different sizes (20 × 20 m; 40 × 40 m; and 60 × 60 m), and none of the quadrats overlapped. Four topographic attributes were calculated for each quadrat of the different cell sizes: elevation, convexity, slope, and aspect. The relative heights at the four corner nodes of each of the 20 × 20 m quadrats, as well as the elevation of the starting node, were measured. Thus, the elevation of a particular 20 × 20 m quadrat could be estimated as the mean of its four corner nodes. The topography of this plot was heterogeneous and rugged, with elevations ranging from 425.3 to 525.8 m above sea level. Following the methods of Yamakura et al. (1995) and Harms, Condit, Hubbell, and Foster (2001), the convexity, slope, and aspect of each quadrat could be calculated utilizing the elevation value (Figure S2; Table S4). To calculate the topographic variables for the other two larger quadrat sizes, the ordinary kriging interpolation method was employed (Legendre & Legendre, 1998). The topographic variables were used as an integrated measure of each quadrat's local environmental conditions, as previous research conducted in this plot has shown that topographic variables have crucial influences on vegetation characteristics (Zhang et al., 2012). A more detailed description of these measurements of the topographic variables can be found in Zhang et al. (2012).

### 2.2 Stand productivity measures

**Figure 1:**
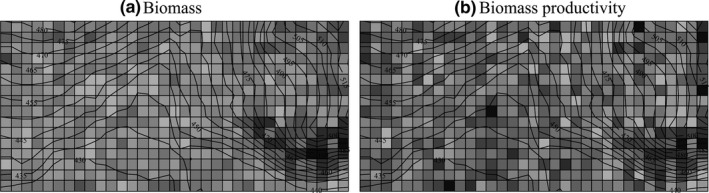
Maps depicting (a) biomass and (b) biomass–productivity patterns at the scale of 20 × 20 m. The shading from light to dark means the observed values from low to high. The lines show the elevation contours at 5 m intervals

All woody plants, including trees and large shrubs with a DBH ≥5 cm in the first census, were included in the analysis. The DBH lower limit was used as the plants above 5 cm DBH were responsible for almost all of the biomass, volume, and productivity. Altogether, 19,911 individuals in 12 families, including 20 genera and 32 species, were encountered within the study plot (Table S1). The average number of species per 20 × 20 m quadrat is 9.59 (range from 4 to 17), per 40 × 40 m quadrat 14.97 (range from 9 to 19) and 60 × 60 m quadrat 18.16 (range from 13 to 23). In this study, the aboveground biomass values (AGB) and volumes (VOL) of individuals were estimated using a set of existing region‐specific allometric equations with DBH as independent variable (Tables S2 and S3). For a given quadrat at different cell sizes, the total AGB and VOL were calculated as the sum of the AGB and VOL of all the individuals within the quadrat, including the individuals that subsequently died. It was found that the average stand AGB and VOL values in 2009 were 95.987 ton/ha and 172.35 m^3^/ha, respectively. The productivity was calculated as the biomass and volume increments (ΔAGB and ΔVOL) from 2009 to 2014 of both surviving individuals and recruits (Figure 1; Figure S3). Specifically, as the existence of a lower cutoff on individual size, the ΔAGB of the recruits were calculated using its actual biomass minus the biomass of the minimum‐sized individual (i.e., individual with a DBH = 5 cm). The possible errors in under‐ or overestimating the species’ AGB and VOL within the quadrats are fairly consistent, and the results are considered to be sufficiently robust (Chisholm et al., 2013).

### 2.3 Functional traits and biodiversity measures

In this study, taxonomic, functional, and phylogenetic diversity are used to evaluate specific biodiversity–productivity relationships. Taxonomic diversity is expressed by the Shannon Index. Functional diversity is measured by a distance‐based functional diversity index: functional dispersion (FDis), which could take account of the relative abundances of the species (Laliberté & Legendre, 2010). A set of plant traits that have been suggested to have great functional significance for plant growth and have been expected to linked with forest productivity were measured (Chiang et al., 2016; Finegan et al., 2015; Liu et al., 2013; Sande et al., 2017). The traits include an architectural trait (maximum height), a stem trait (wood density), and six leaf traits: leaf area, specific leaf area, leaf dry matter content, leaf carbon concentration, leaf nitrogen concentration, and leaf carbon–nitrogen ratio (Table 1). All functional traits were determined for 32 woody species. Maximum height was measured using an altimeter pole together with a laser telemeter (TruPulse360, Laser Technology Inc., USA). Wood and leaf traits were collected from 10 to 30 individuals for each species. Wood cores were extracted from the cortex to the pith at 1.3 m height using an increment borer (5 mm, Suunto, Finland). Wood density was determined by dividing the wood core dry weight (80°C, 72 hr) by its fresh volume (Williamson & Wiemann, 2010). Leaf traits were measured on individuals with DBH between 10 and 20 cm. At least five fresh leaf samples were taken from each individual on the highest parts of the tree crown, which were fully exposed to direct sunlight (Liu et al., 2013). Leaf area, leaf dry matter content (leaf dry mass/leaf fresh mass), and specific leaf area (leaf area/dry matter) were obtained using standard methods (Cornelissen et al., 2003). Leaf carbon and nitrogen concentrations were gathered using an elemental analyzer (PE2400 SeriesII, PerkinElmer Inc., USA). Leaf carbon–nitrogen ratios were calculated by dividing the leaf carbon concentrations by the leaf nitrogen concentrations. Based on these plant traits, FDis could be calculated. FDis was defined as the mean distance in the multidimensional trait space of individual species to the centroid of all species in the community (Laliberté & Legendre, 2010) which expresses the degree of the trait dissimilarities among the species and may increase the ways in which species are able to access and utilize resources (Chiang et al., 2016). Prior to the calculation, all of the trait data were rescaled to center on 0 with a standard deviation of 1 in order to eliminate the effects of the dimensions and magnitudes of the data (Villéger et al., 2008). Besides, to eliminate the correlation of traits, a principal coordinates analysis (PCoA) was first performed on the species–traits matrix, then the resulting PCoA axes were used as the new “traits” together with a species–abundance matrix to compute the FDis (Laliberté & Legendre, 2010; Villéger et al., 2008).

**Table 1:** Functional traits and their significance

Functional traits	Unit	Functional significance
Leaf area (LA)	mm^2^	Light acquisition
Specific leaf area (SLA)	mm^2^/g	Leaf economic spectrum; photosynthetic potential; plant shade tolerance
Leaf dry matter content (LDMC)	mg/g	Leaf water relations; predictor of species conservatism
Leaf carbon concentration (LC)	mg/g	Carbon assimilation rate
Leaf nitrogen concentration (LN)	mg/g	Leaf economic spectrum; photosynthetic potential; nitrogen acquisition
Leaf carbon–nitrogen ratio (C/N)	%	Trade‐off between leaf carbon and nutrient investment
Wood density (WD)	g/mm^3^	Wood economic spectrum; trade‐off between growth and survival; water transport and allocation
Maximum height (Hmax)	m	Plant competitive vigor and strategy; light niche; structural diversity

**Figure 2:**
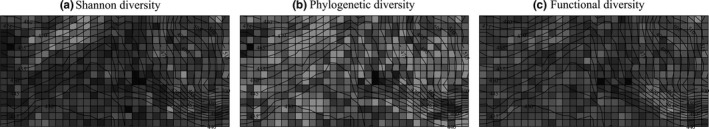
Maps depicting (a) Shannon, (b) phylogenetic, and (c) functional diversity patterns at the scale of 20 × 20 m. The shading from light to dark means the observed values from low to high. The lines show the elevation contours at 5 m intervals

The third measure of diversity is Faith's phylogenetic diversity index (PD), which is based on the species’ evolutionary distances. A phylogenetic tree which includes 32 species in the study area was constructed using an informatics tool named Phylomatic. Phylomatic utilizes the phylogeny of Angiosperm Phylogeny Group III as a backbone (Webb & Donoghue, 2005). The branch lengths were estimated for this “super‐tree” based on the time of the angiosperm‐wide divergence. Undated nodes were interpolated using the algorithm of the branch length adjustment (BLADJ) in the Phylocom software (Webb, Ackerly, & Kembel, 2008). The PD was calculated as the sum of the branch lengths for the species present in a particular quadrat. As a consequence, the PD was influenced not only by the species richness, but also by how closely species were related to each other (Cadotte, et al., 2009; Srivastava et al.,^2012^). Finally, as the relationships between the biodiversity and forest productivity may be scale‐dependent, we calculated the three diversity indices for each of the different cell sizes (Figure 2; Figure S4).

### 2.4 Statistical analysis

**Figure 3:**
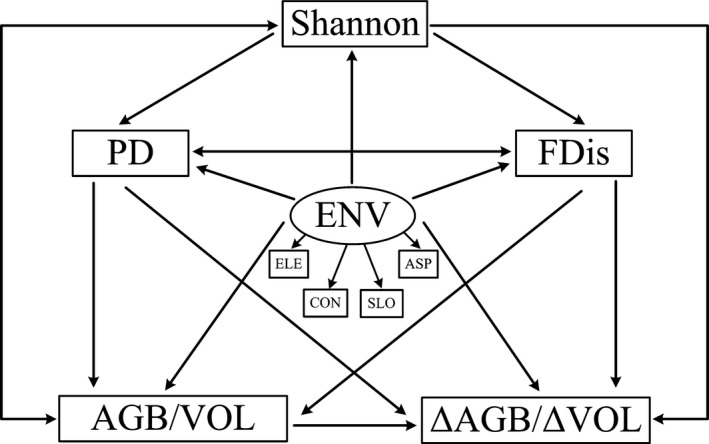
Metamodel of the structural equation employed to explore the complicated relationships: The arrows represent the hypothesized causal relationships between the variables; ENV represents the environment latent variable; ELE is the elevation; CON refers to the convexity; SLO is the slope; ASP represents the aspect; Shannon is the Shannon species diversity index; PD is the Faith's phylogenetic diversity index; FDis represents the functional dispersion index; AGB is the aboveground biomass; ΔAGB represents the average annual AGB increment; VOL is the stand volume; and ΔVOL is the average annual VOL increment

In order to elucidate the direct and indirect causal relationships between the biodiversity and forest productivity at different spatial scales, SEMs were employed. SEMs estimate the path coefficients and the variations of the different variables and need an a priori hypothesis (Ali et al., 2016; Grace et al., 2012). Thus, a metamodel based on the known theoretical causal relations between biodiversity and productivity was constructed. The influences of the initial stand volume or biomass, as well as the effects of the environmental factors, were simultaneously accounted for, as shown in Figure 3. In this model, the environmental conditions were treated as a latent variable by incorporating the four variables elevation, convexity, slope, and aspect. We hypothesized that: (i) The environmental conditions play a fundamental role in determining the biotic factors; (ii) the stand volume, biomass, and productivity are directly influenced by biodiversity; (iii) the FDis and PD will be affected by species diversity; in addition, the FDis and PD are correlated; (iv) the initial stand volume and biomass will directly affect forest productivity. To increase the interpretation of the results and to test the edge effects from neighboring quadrats, we constructed a nested model, in which FD and PD were calculated including all individuals in the 20 × 20 m quadrats, whereas biomass and productivity were calculated for the inner individuals with a 5‐m buffer from the quadrat margin (Tobner et al., 2016). We used the chi‐square difference test to compute the difference between the two models (Rosseel, 2012).

The SEMs were fitted using a maximum likelihood approach and evaluated using the Bentler's comparative fit index (CFI) and standardized root mean square residual (SRMR), as recommended by Hoyle (2012). The cutoff values of the goodness‐of‐fit were CFI >0.9 and SRMR <0.08. Prior to the SEM analysis, all the observations of the different variables were rescaled to center on 0 with a standard deviation of 1, in order to alleviate departures from normality and to make the ranges of all variables comparable in a similar scale so that fitting the SEM is made possible. In addition, the environmental data were first square‐root transformed (Grace, Anderson, Olff, & Scheiner, 2010). Finally, for the purpose of increasing the contrast and interpretation of the results, the bivariate relationships between the biodiversity and productivity were simultaneously examined using simple linear regression models. The SEMs were implemented using the *lavaan* package (Rosseel, 2012) in R 3.3.2 (http://www.r-project.org).

## 3 RESULTS

**Figure 4:**
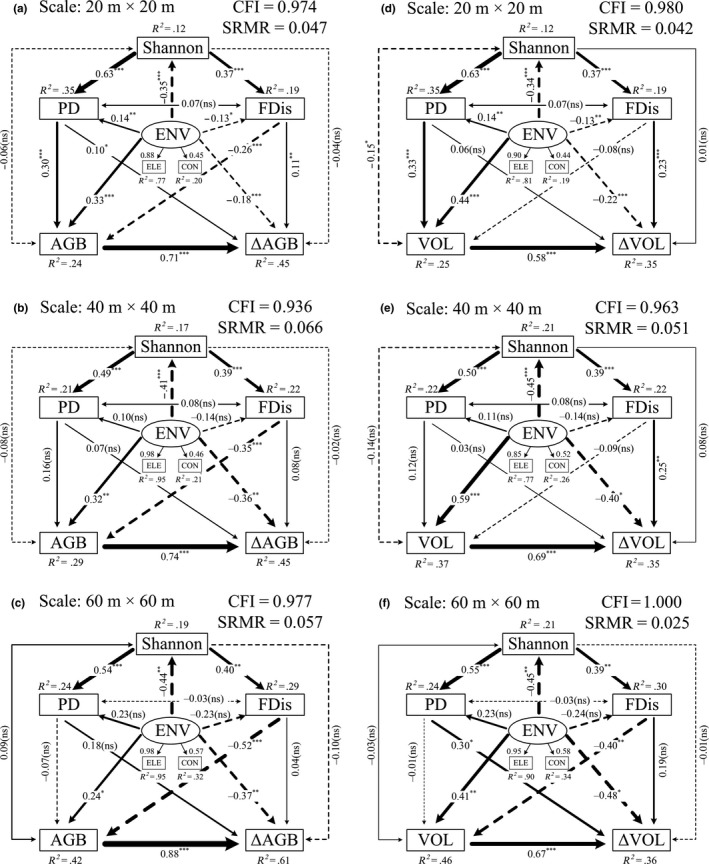
Results of the structural equation models’ (SEMs) analysis for the effects of the local environmental conditions, biodiversity, and stand attributes (represented by the stand AGB or VOL) on: (a) ΔAGB at the spatial scale of 20 × 20 m; (b) ΔAGB at the spatial scale of 40 × 40 m; (c) ΔAGB at the spatial scale of 60 × 60 m; (d) ΔVOL at the spatial scale of 20 × 20 m; (e) ΔVOL at the spatial scale of 40 × 40 m; and (f) ΔVOL at the spatial scale of 60 × 60 m. The arrows represent the hypothesized causal relationships between the variables. The solid lines represent the positive relationships, and the dashed lines represent the negative relationships. The values next to the arrows are the standardized path coefficients with corresponding statistical significance (***p <* *.001; **p <* *.01; *p <* *.05; ns, nonsignificant). The line width is proportional to the standardized path coefficient. The values of *R* ^2^ represent the percentage of the response variations explained by the observed variable. The variable abbreviations are the same as shown in Figure 3

The SEMs for the complex relationships between the biodiversity and productivity conformed well to the observations (CFI = 0.936–1.000; SRMR = 0.025–0.066), as shown in Figure 4. At the 20 × 20 m scale, 45% of the variation in the ΔAGB was accounted for by the explanatory variables (Figure 4a). Among the three components of the biodiversity, the PD and FDis had significant direct positive effects on the ΔAGB (standardized path coefficient, *r* = .10 and .11, respectively). Shannon was found to have no significant direct effect on ΔAGB. ΔAGB increased with the initial AGB of the stand, and its standardized path coefficient showed the largest value (*r* = .71) in this specific SEM. At the same spatial scale, 24% of the variation in the AGB could be explained by the biodiversity and environmental conditions. The PD and FDis were found to have significant direct effects on AGB, while the effect of Shannon was found to be insignificant. The effect of PD on AGB was positive (*r* = .30), but the effect of the FDis was negative (*r* = −.26). Therefore, it was concluded that PD had significant indirect positive effect on ΔAGB mediated by the AGB (*r* = .21). While the indirect effect of the FDis via AGB was negative (*r* = −.18). When these results were taken together, the total effects (direct and indirect) of PD were determined to be significantly positive. However, the total effects of the FDis were canceled out and negligible (Figure 4a; Table 2). Although significant direct effect of Shannon on the ΔAGB was not found, the indirect effects of Shannon on ΔAGB were significantly positive through the positive effects on PD (*r* = .06) and FDis (*r* = .04), as both PD and FDis were strongly increasing with increasing Shannon (Figure 4a; Table 2).

**Table 2:** Direct, indirect, and total standardized effects on the forest productivity at different spatial scales, based on the structural equation models

Predictor	Pathway	ΔAGB			ΔVOL		
		20 × 20 m	40 × 40 m	60 × 60 m	20 × 20 m	40 × 40 m	60 × 60 m
ENV	Direct	−**0.176**	−**0.358**	−**0.372**	−**0.219**	−**0.399**	−**0.483**
	Indirect through Shannon	0.015	0.007	0.048	−0.005	−0.038	0.014
	Indirect through PD	0.015	0.007	0.042	0.008	0.003	0.070
	Indirect through FDis	−**0.015**	−0.011	−0.008	−**0.031**	−0.036	−0.046
	Indirect through AGB or VOL	**0.235**	**0.240**	0.216	**0.256**	**0.392**	**0.277**
	Total	0.074	−0.115	−0.074	−0.011	−0.077	−0.168
Shannon	Direct	−0.043	−0.017	−0.109	0.014	0.083	−0.030
	Indirect through PD	**0.064**	0.036	0.099	0.038	0.014	0.164
	Indirect through FDis	**0.041**	0.030	0.014	**0.085**	**0.096**	0.074
	Indirect through AGB or VOL	−0.043	−0.062	0.083	−**0.089**	−0.091	0.017
	Total	0.019	−0.012	0.086	0.049	0.102	0.225
PD	Direct	**0.103**	0.074	0.183	0.061	0.027	**0.301**
	Indirect through AGB or VOL	**0.208**	0.120	−0.058	**0.190**	0.078	−0.003
	Total	**0.311**	**0.194**	0.125	**0.251**	0.105	**0.298**
FDis	Direct	**0.110**	0.077	0.035	**0.232**	**0.250**	0.190
	Indirect through AGB or VOL	−**0.181**	−**0.261**	−**0.459**	−0.048	−0.057	−**0.226**
	Total	−0.071	−0.189	−**0.423**	**0.183**	**0.193**	−0.075
VOL	Direct	**0.707**	**0.742**	**0.884**	**0.584**	**0.670**	**0.670**

The standardized coefficients in bold fonts mean that the effects are significant at the level of 0.05. The variable abbreviations are the same as shown in Figure 3.

At the aforementioned spatial scale, it was found that the results of the SEM based on the ΔVOL did not correspond very well with the results of ΔAGB (Figure 4d). In the ΔVOL model, a significant relation between PD and ΔVOL was not found. Additionally, the influence of the FDis on VOL was found to be insignificant. However, Shannon unexpectedly displayed a significantly negative effect on VOL (*r* = −.15). Therefore, in this model, based on the reasons outlined above, the Shannon was able to exert an indirect impact on ΔVOL through the VOL (*r* = −.09). However, its indirect impact which was mediated by PD had vanished. The indirect impact of the FDis on the ΔVOL via VOL had also disappeared. On the other hand, although the goodness‐of‐fit of this model was slightly higher than that of the ΔAGB model, only 35% of the variation was explained when compared with the 46% of the ΔAGB model. Table 2 presents more detailed information regarding the direct and indirect effects of the explanatory variables on forest productivity.

At the two larger spatial scales, although the SEMs also were well supported by the data, the biodiversity–productivity relationships were not found to be as significant as in the 20 × 20 m scale (Figure 4). For example, according to the ΔAGB models, there were no significant relationships between biodiversity and productivity at the two larger scales (Figure 4b,c). For the ΔVOL models, ΔVOL increased with increasing FDis at the 40 × 40 m scale (*r* = .25; Figure 4e) and increased with increasing PD at the 60 × 60 m scale (*r* = .30; Figure 4f). The variation of the ΔAGB was better accounted for by the explanatory variables than those of the ΔVOL, as expressed by the greater *R* ^2^ values. Surprisingly, no obvious connection was found between the PD and FDis in each of the SEMs, despite the fact that they were both increasing with greater Shannon values. The environmental conditions were represented by elevation and convexity in these SEMs. The other two topographic variables were excluded in the best‐fitted SEMs. At the 20 × 20 m scale, the environmental conditions had significant direct effects on all biotic factors, including the three facets of biodiversity and forest productivity. With increasing spatial scale, the influence of environmental conditions on PD and FDis became less prominent. However, the influence on Shannon, AGB, VOL, and productivity remained remarkably high.

In order to improve the interpretation of the results and to test the edge effects from neighboring quadrats, the chi‐square difference test was used to compare the two nested models that with a five‐meter buffer or not. We found that there is no significant difference between the two nested models (Figure S5). Meanwhile, the results which were based on the simple bivariate analyses and SEMs were compared, revealing several differences (Figures S6; S7; S8; and S9). At the 20 × 20 m scale, the bivariate relationships showed that the ΔVOL increased with Shannon (Figure S7), while this tendency did not emerge in the corresponding SEM (Table 2). At the 40 × 40 m scale, the effect of the PD on the ΔAGB was significantly positive in SEM (Table 2), while in the bivariate analyses, there was no apparent association between PD and ΔAGB (Figure S7). The other relationships estimated in the bivariate analyses were almost consistent with the total effects in the SEM.

## 4 DISCUSSION

In this study, we seek to evaluate the relative importance of different components of biodiversity, simultaneously including the effects of environmental conditions and stand maturity at varying spatial scales. The results show that the PD and FDis are more closely related to forest productivity when compared with the species diversity (Shannon). In addition, based on the results of the SEMs, several mechanisms were detected which could not be found based on the bivariate analyses.

### 4.1 Scale‐dependent relationships between biodiversity and productivity

At the 20 × 20 m scale, we found that the PD and FDis had significant effects on forest productivity. In contrast, species diversity had no direct effects on productivity, but was only mediated by PD and FDis. The FDis measures the functional dissimilarity regarding the species’ competitive ability, resource access strategy, and the trade‐off between growth and survival (Laliberté & Legendre, 2010). The PD measures the species’ evolutionary distance (Cadotte et al., 2008). It is generally acknowledged that evolutionary dissimilarity may generate trait dissimilarity (Cadotte et al., 2008; Liu et al., 2013). However, based on the SEMs, we found that there was no obviously direct connection between the PD and FDis. This result was not surprising, because the assumption that PD is connected with FDis will only be tenable when the selected traits are conserved over the phylogeny (Flynn et al., 2011). Therefore, we could not conclude that PD was not an inefficient measurement of ecosystem functioning. On the other hand, although there were eight traits selected in our analyses, it is obvious that this selection is incomplete in representing the total species function. Several unmeasured traits, for example, biological nitrogen fixation and pathogen tolerance, may have been conserved in the phylogeny (Petermann, Fergus, Turnbull, & Schmid, 2008). Flynn et al. (2011) concluded that both the community trait dissimilarity and the evolutionary history can be valuable predictors of an ecosystem's function, although the trait dissimilarity was only partially related to phylogenetic distances. This is consistent with our results.

The niche complementarity effect hypothesis states that a diverse group of species has a greater variety of traits and allows species to reduce interspecific competition and better utilize a pool of limiting resources, thereby increasing total ecosystem productivity, than a less diverse community. In previous studies, PD and FDis were used to assist in the understanding of how biodiversity relates to niche complementarity effects (Cadotte et al., 2008, 2010; Flynn et al., 2011; Laliberté & Legendre, 2010). Positive relationships between biodiversity and productivity were detected in our research, which supports the hypothesis that complementarity effects can play an important role in forest ecosystems. However, at greater spatial scales, the results may be different. At the 40 × 40 m and 60 × 60 m scales, the relationships between biodiversity and productivity are only weakly correlated. For example, not only was the influence of the species diversity insignificant, but the effects of PD and FDis were also found to be reduced.

We find that the forest biodiversity–productivity relationships are scale‐dependent. At the smaller scales, functional or phylogenetic diversity plays a significant role in determining productivity. However, with increasing quadrat size, the proportion of species with similar functional traits increases, resulting in greater functional overlap or functional redundancy (Dalerum, Cameron, Kunkel, & Somers, 2012; Loreau, 2004). Functional redundancy refers to different species having similar functional traits and utilizing nearly identical resources within a community (Dalerum et al., 2012; Loreau, 2004). Thus, when species diversity reaches a certain degree, the effects of complementarity and facilitation will reach a plateau (Lohbeck et al., 2012). Under those circumstances, the changes in diversity will no long affect ecosystem productivity (Loreau, 2004). It is generally believed that it is easier to reach a saturation of resource utilization in the tropical forest with high species diversity. However, our findings show that in a temperate forest with a relatively low species diversity, functional redundancy may also exist at the greater spatial scales.

### 4.2 Environmental conditions determine forest performances

Previous studies have shown that the relationships between biodiversity and productivity are regulated, directly and indirectly, by a large number of factors (Liu et al., 2016). However, to our knowledge, few (if any) studies have yet been able to integrate these factors into a research framework which simultaneously includes the different components of biodiversity, productivity, stand maturity, and environmental conditions, especially regarding a temperate forest. In this study, SEMs were employed to evaluate the complex multivariate causality of biodiversity and productivity, including various other factors.

The results of this study have confirmed the fundamental roles of environmental conditions in determining ecosystem performance in terms of biodiversity, stand maturity, and productivity. It was found that, at the smallest scale Shannon, PD and FDis were all affected by the environmental conditions. However, at slightly greater scales, these influences on PD and FDis disappeared. Such changes could be attributed to the small‐scale habitat heterogeneity, which has been found to considerably shape tree species diversity and distribution (Healy et al., 2008; Liu et al., 2014). It appears that the habitat specificity decreases with increasing quadrat size. Therefore, the selection or filtration of environmental factors on plant traits also decreased. Hooper et al. (2005) and Healy et al. (2008) concluded that the effects of biodiversity on productivity depend on their interactions with the environment, because the environmental conditions may influence the species’ complementarity. These findings could provide insights regarding how habitat heterogeneity regulates biodiversity effects at different spatial scales.

The direct path of SEMs showed that forest productivity decreased with increasing altitude and convexity, probably as a result of poorer soil moisture and nutrient conditions in the habitats with relatively higher altitude and greater convexity. However, it should be noted that the effects of the environmental conditions on forest productivity were also indirectly explained by the stand maturity. As a consequence, the combined effects of the environmental conditions on the productivity of the forest were partly neutralized. In the SEMs, the standardized path coefficient of the stand maturity relating to productivity was almost consistently greatest in each of the models. Accordingly, the stand maturity expressed by the initial AGB or VOL should be regarded as the most crucial endogenous driver of forest productivity. In this context, Vilà et al. (2007) concluded that the positive stand biomass–productivity correlation could be regarded as an indication of an early forest seral stage. This finding is consistent with the fact that our study plot is a near‐mature forest where the last recorded tree harvesting activities had taken place approximately 50 years ago (Zhang et al., 2012).

### 4.3 Increment of woody biomass is a better proxy of productivity

In a number of previous studies, volume production was used to measure forest productivity (Gadow & Hui, 1999; Liang et al., 2016). Forest volume is an important target variable assessed most national forest surveys (Bettinger et al., 2010). However, in current studies, researchers have argued that volume should not be regarded as the best measure of productivity, as stand volume ignores the differences in wood density and only contains the merchantable stem‐wood portion (Russell et al., 2014). The biomass considers the differences in wood density, as well as other woody components (Russell et al., 2014). In this study, the results of the SEMs were compared regarding ΔAGB and ΔVOL. It was found that ΔAGB was a better predictor than ΔVOL. The biodiversity–productivity relationships based on the SEMs of ΔVOL seemed to be less consistent. At the 20 × 20 m and 40 × 40 m scales, ΔVOL increased with the FDis, while the effect of PD was insignificant. However, at the 60 × 60 scale, the influence of the FDis had vanished, while ΔVOL positively correlated with PD. It was very difficult to find a reasonable ecological explanation for this unexpected phenomenon. However, in the case of ΔAGB, the results were more robust and consistent. In addition, the ΔAGB variation was better accounted for by the explanatory variables than that of ΔVOL, which was reflected by the greater *R* ^2^ values. Therefore, we conclude that ΔAGB is a better proxy of forest productivity, if it can be estimated with sufficient accuracy. However, estimates of biomass are more difficult to obtain than estimates of volume, especially at large geographic scales, due to the environmental plasticity of wood densities (Lintunen & Kaitaniemi, 2010; Osada, Tateno, Mori, & Takeda, 2004; Sapijanskas, Paquette, Potvin, Kunert, & Loreau, 2014). Skovsgaard and Vanclay (2008) suggested that the most suitable measure may depend on the scale and purpose of the research. Stand volume production is of economic importance and therefore preferred by forest managers.

## 5 CONCLUSIONS

The results in our temperate forest show that the biodiversity–productivity relationships are scale‐dependent. The positive role of biodiversity in facilitating forest productivity is confirmed, but only at small scales. The relations between forest biodiversity and productivity are influenced by a number of biotic and abiotic factors, such as stand maturity and various local environmental conditions, and we show that simple bivariate analyses are insufficient to untangle this complexity. The specific roles of the different influencing factors may differ significantly, depending on the spatial scale. We expect that the findings of this study will assist in achieving a better understanding of the complex relationships between biodiversity and productivity in temperate forest ecosystems.

## CONFLICT OF INTERESTS

None declared.

## AUTHORS’ CONTRIBUTIONS

MinHui Hao analyzed the data and wrote the manuscript; Xiuhai Zhao and Klaus von Gadow modeled and interpreted the results; Chunyu Zhang initiated the research and performed project coordination.
