# Supplementary material for: Functional and phylogenetic diversity determine woody productivity in a temperate forest
Source: Ecol Evol. 2018 Jan 29;8(5):2395–406. doi: 10.1002/ece3.3857 (PMC5838064; doi:10.1002/ece3.3857)
Supplement: Supplementary file 1 [file ECE3-8-2395-s001.docx]

**TABLE S1** Species information in the permanent forest plot. Based on the APG Ⅲ classification system.

| No. | Species | Genues | Family |
| --- | --- | --- | --- |
| 1 | *Betula costata* | *Betula* | Betulaceae |
| 2 | *Betula platyphylla* | *Betula* | Betulaceae |
| 3 | *Carpinus cordata* | *Carpinus* | Betulaceae |
| 4 | *Maackia amurensis* | *Maackia* | Fabaceae |
| 5 | *Quercus mongolica* | *Quercus* | Fagaceae |
| 6 | *Juglans mandshurica* | *Juglans* | Juglandaceae |
| 7 | *Tilia amurensis* | *Tilia* | Malvaceae |
| 8 | *Tilia* *mandshurica* | *Tilia* | Malvaceae |
| 9 | *Fraxinus mandchurica* | *Fraxinus* | Oleaceae |
| 10 | *Fraxinus rhynchophylla* | *Fraxinus* | Oleaceae |
| 11 | *Syringe reticulata* | *Syringe* | Oleaceae |
| 12 | *Abies holophylla* | *Abies* | Pinaceae |
| 13 | *Pinus koraiensis* | *Pinus* | Pinaceae |
| 14 | *Cerasus maximowiczii* | *Cerasus* | Rosaceae |
| 15 | *Malus baccata* | *Malus* | Rosaceae |
| 16 | *Padus racemose* | *Padus* | Rosaceae |
| 17 | *Sorbus alnifolia* | *Sorbus* | Rosaceae |
| 18 | *Sorbus dacica* | *Sorbus* | Rosaceae |
| 19 | *Rhamnus* *davurica* | *Rhamnus* | Rhamnaceae |
| 20 | *Phellodendron amurense* | *Phellodendron* | Rutaceae |
| 21 | *Populous davidiana* | *Populous* | Salicaceae |
| 22 | *Populous ussuriensis* | *Populous* | Salicaceae |
| 23 | *Salix rorida* | *Salix* | Salicaceae |
| 24 | *Acer barbinerve* | *Acer* | Sapindaceae |
| 25 | *Acer ginnala* | *Acer* | Sapindaceae |
| 26 | *Acer mono* | *Acer* | Sapindaceae |
| 27 | *Acer* *mandshuricum* | *Acer* | Sapindaceae |
| 28 | *Acer tegmentosum* | *Acer* | Sapindaceae |
| 29 | *Acer triflorum* | *Acer* | Sapindaceae |
| 30 | *Ulmus japonica* | *Ulmus* | Ulmaceae |
| 31 | *Ulmus macrocarpa* | *Ulmus* | Ulmaceae |
| 32 | *Ulmus laciniata* | *Ulmus* | Ulmaceae |

**TABLE S2** Region-specific allometric equations for the calculation of above-ground biomass for each tree species. AGB represents the above-ground biomass (in kg); DBH refers to the diameters at breast height (in cm).

| No. | Species | Allometric equations | Similar species |
| --- | --- | --- | --- |
| 1 | *Abies holophylla* | AGB=exp(-2.989+2.613×ln(DBH)) |  |
| 2 | *Acer mandshuricum* | AGB=exp(-2.111+2.310×ln(DBH)) | *Acer barbinerve*  *Acer ginnala*  *Acer tegmentosum*  *Acer triflorum* |
| 3 | *Acer mono* | AGB=exp(-2.164+2.336×ln(DBH)) |  |
| 4 | *Betula platyphylla* | AGB=exp(-1.941+2.286×ln(DBH)) | *Betula costata* |
| 5 | *Carpinus cordata* | AGB=exp(-1.909+2.111×ln(DBH)) |  |
| 6 | *Fraxinus mandschurica* | AGB=exp(-2.301+2.443×ln(DBH)) | *Fraxinus rhynchophylla*  *Phellodendron amurense* |
| 7 | *Juglans mandshurica* | AGB=exp(-2.466+2.381×ln(DBH)) |  |
| 8 | *Maackia amurensis* | AGB=exp(-2.001+2.198×ln(DBH)) | *Cerasus maximowiczii*  *Malus baccata*  *Padus racemose*  *Rhamnus davurica*  *Syringe reticulate* |
| 9 | *Pinus koraiensis* | AGB=exp(-3.394+2.582×ln(DBH)) |  |
| 10 | *Populous ussuriensis* | AGB=exp(-2.507+2.358×ln(DBH)) | *Populous davidiana*  *Salix rorida* |
| 11 | *Quercus mongolica* | AGB=exp(-2.797+2.571×ln(DBH)) |  |
| 12 | *Tilia amurensis* | AGB=exp(-2.364+2.323×ln(DBH)) | *Tilia mandshurica* |
| 13 | *Ulmus japonica* | AGB=exp(-2.058+2.271×ln(DBH)) | *Ulmus macrocarpa*  *Ulmus laciniata*  *Sorbus dacica*  *Sorbus alnifolia* |

**TABLE S3** Allometric equations for the calculation of stem volume for each tree species. Based on the Region-Specific Volume Table of Jiaohe in Jilin province (The forestry department of jilin province). VOL represents the stem volume (in m^3^); DBH refers to the diameters at breast height (in cm).

| No. | Allometric equations | Species involved |
| --- | --- | --- |
| 1 | VOL=5.507×10^-5^×DBH^1.77206161×(39.2601-1103.6355/(DBH+27))^1.10349762 | *Abies holophylla* |
| 2 | VOL=5.085×10^-5^×DBH^1.80946002×(48.9089-2409.1698/( DBH+50))^1.10143150 | *Pinus koraiensis* |
| 3 | VOL=4.071×10^-5^×DBH^1.71904224×(24.5254-247.3559/( DBH+8))^1.25350526 | *Quercus mongolica* |
| 4 | VOL=3.545×10^-5^×DBH^1.76789775×(28.4117-400.0149/( DBH+13))^1.24396035 | *Betula costata*  *Tilia amurensis*  *Tilia mandshurica* |
| 5 | VOL=4.061×10^-5^×DBH^1.83543102×(30.7553-452.8747/( DBH+14))^1.11310324 | *Betula platyphylla*  *Populous davidiana*  *Populous ussuriensis*  *Salix rorida* |
| 6 | VOL=5.129×10^-5^×DBH^1.93014641×(29.7096-447.2660/( DBH+14))^0.93487618 | *Fraxinus mandschurica*  *Fraxinus rhynchophylla*  *Juglans mandshurica*  *Phellodendron amurense* |
| 7 | VOL=3.629×10^-5^×DBH^1.81966905×(27.0143-477.2749/( DBH+18))^1.17303013 | *Acer barbinerve*  *Acer ginnala*  *Acer mandshuricum*  *Acer mono*  *Acer tegmentosum*  *Acer triflorum*  *Ulmus laciniata*  *Ulmus macrocarpa*  *Ulmus japonica* |
| 8 | VOL=4.251×10^-5^×DBH^1.78308888×(22.9614-263.2795/( DBH+11))^1.14082685 | *Carpinus cordata*  *Cerasus maximowiczii*  *Malus baccata*  *Maackia amurensis*  *Padus racemose*  *Rhamnus davurica*  *Syringe reticulate*  *Sorbus dacica*  *Sorbus alnifolia* |

**TABLE S4** Pearson correlation coefficients for pairwise correlations between topographic variable.

| Topographic variable | | Elevation | Convexity | Aspect |
| --- | --- | --- | --- | --- |
| 20 × 20 m | Convexity | 0.442^***^ |  |  |
|  | Aspect | 0.441^***^ | 0.084 |  |
|  | Slope | 0.422^***^ | 0.172^***^ | 0.056 |
| 40 × 40 m | Convexity | 0.440^***^ |  |  |
|  | Aspect | 0.463^***^ | 0.017 |  |
|  | Slope | 0.458^***^ | 0.207^*^ | 0.101 |
| 60 × 60 m | Convexity | 0.504^***^ |  |  |
|  | Aspect | 0.529^***^ | 0.263 |  |
|  | Slope | 0.471^***^ | 0.082 | 0.091 |

Note: *** p<0.001; ** p<0.01; * p<0.05.

**FIGURE S1** Map of China showing the location of the 21.12 ha forest permanent plot.

**FIGURE S2** Maps depicting four topographic variables at the scale of 20 × 20 m: a) Elevation, b) Convexity, c) Aspect and d) Slope. The shading from light to dark means the observed values from low to high. The lines show the elevation contours at 5 m intervals.

.

**FIGURE S3** Maps depicting a) biomass and b) biomass productivity patterns at the scale of 40 × 40 m, c) biomass and d) biomass productivity patterns at the scale of 60 × 60 m,. The shading from light to dark means the observed values from low to high. The lines show the elevation contours at 5 m intervals.

**FIGURE S4** Maps depicting a) Shannon, b) Phylogenetic and c) Functional diversity patterns at the scale of 40 × 40 m, d) Shannon, e) Phylogenetic and f) Functional diversity patterns at the scale of 60 × 60 m. The shading from light to dark means the observed values from low to high. The lines show the elevation contours at 5 m intervals.

**FIGURE S5** Results of Chi Square Difference Test between two nested SEM models: a) SEM with a five meters buffer and b) SEM with no buffer at the scale of 20 m × 20 m. The Chi Square Difference of the two nested SEM models is 6.956 (*p*=1) indicating that there is no significant difference between the two models. ENV represents the environment latent variable; ELE is the elevation; CON refers to the convexity; Shannon is the Shannon species diversity index; PD is the Faith’s phylogenetic diversity index; FDis represents the functional dispersion index; AGB is the aboveground biomass; ΔAGB represents the average annual AGB increment.

**FIGURE S6** Simple bivariate analyses between biodiversity and the initial biomass (or volume). a) AGB at the scale of 20 × 20 m; b) VOL at the scale of 20 × 20 m; c) AGB at the scale of 40 × 40 m; d) VOL at the scale of 40 × 40 m; e) AGB at the scale of 60 × 60 m; and f) VOL at the scale of 60 × 60 m. The significant fitted relationships at *p* < 0.05 are shown in the plot. The variable abbreviations are the same as shown in Figure S5.

**FIGURE S7** Simple bivariate analyses between biodiversity and forest productivity. a) ΔAGB at the scale of 20 × 20 m; b) ΔVOL at the scale of 20 × 20 m; c) ΔAGB at the scale of 40 × 40 m; d) ΔVOL at the scale of 40 × 40 m; e) ΔAGB at the scale of 60 × 60 m; and f) ΔVOL at the scale of 60 × 60 m. The significant fitted relationships at *p* < 0.05 are shown in the plot. The variable abbreviations are the same as shown in Figure S5.

**FIGURE S8** Simple bivariate analyses between four topographic variables and the initial biomass (or volume). a) AGB at the scale of 20 × 20 m; b) VOL at the scale of 20 × 20 m; c) AGB at the scale of 40 × 40 m; d) VOL at the scale of 40 × 40 m; e) AGB at the scale of 60 × 60 m; and f) VOL at the scale of 60 × 60 m. The significant fitted relationships at *p* < 0.05 are shown in the plot. The variable abbreviations are the same as shown in figure S5.

**FIGURE S9** Simple bivariate analyses between four topographic variables and forest productivity. a) ΔAGB at the scale of 20 × 20 m; b) ΔVOL at the scale of 20 × 20 m; c) ΔAGB at the scale of 40 × 40 m; d) ΔVOL at the scale of 40 × 40 m; e) ΔAGB at the scale of 60 × 60 m; and f) ΔVOL at the scale of 60 × 60 m. The significant fitted relationships at *p* < 0.05 are shown in the plot. The variable abbreviations are the same as shown in figure S5.
